# Electrolyte‐Gated Vertical Synapse Array based on Van Der Waals Heterostructure for Parallel Computing

**DOI:** 10.1002/advs.202103808

**Published:** 2021-12-26

**Authors:** Seyong Oh, Ju‐Hee Lee, Seunghwan Seo, Hyongsuk Choo, Dongyoung Lee, Jeong‐Ick Cho, Jin‐Hong Park

**Affiliations:** ^1^ Department of Electrical and Computer Engineering Sungkyunkwan University Suwon 16419 Korea; ^2^ Sungkyunkwan Advanced Institute of Nanotechnology (SAINT) Sungkyunkwan University Suwon 16417 Korea

**Keywords:** hardware artificial neural networks, ion gel, parallel computing, synapse arrays, van der Waals heterostructures, vertical synaptic devices

## Abstract

Recently, three‐terminal synaptic devices, which separate read and write terminals, have attracted significant attention because they enable nondestructive read‐out and parallel‐access for updating synaptic weights. However, owing to their structural features, it is difficult to address the relatively high device density compared with two‐terminal synaptic devices. In this study, a vertical synaptic device featuring remotely controllable weight updates via e‐field‐dependent movement of mobile ions in the ion‐gel layer is developed. This synaptic device successfully demonstrates all essential synaptic characteristics, such as excitatory/inhibitory postsynaptic current (E/IPSC), paired‐pulse facilitation (PPF), and long‐term potentiation/depression (LTP/D) by electrical measurements, and exhibits competitive LTP/D characteristics with a dynamic range (*G*_max_/*G*_min_) of 31.3, and asymmetry (AS) of 8.56. The stability of the LTP/D characteristics is also verified through repeated measurements over 50 cycles; the relative standard deviations (RSDs) of *G*_max_/*G*_min_ and AS are calculated as 1.65% and 0.25%, respectively. These excellent synaptic properties enable a recognition rate of ≈99% in the training and inference tasks for acoustic and emotional information patterns. This study is expected to be an important foundation for the realization of future parallel computing networks for energy‐efficient and high‐speed data processing.

## Graphical abstract

New type of vertical synapse array with long‐term plasticity is implemented by using van der Waals heterostructure and ion‐gel weight control layer. Important synaptic properties including long‐term potentiation/depression are analyzed through electrical measurements, and the applicability of the vertical synaptic device to artificial neural networks is demonstrated via training and inference tasks for acoustic and emotional information patterns.

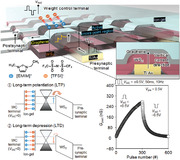

## 1 Introduction

Parallel computing, which can provide energy‐efficient and high‐speed data processing, has recently attracted considerable attention as a future technology that can deal with a tremendous amount of unstructured data such as images, text, sound, and video.^[^ ^1^, ^2^, ^3^, ^4^, ^5^, ^6^ ^]^ One of the well‐researched parallel computing technologies is brain‐inspired neuromorphic computing, which is based on hardware artificial neural networks (HW‐ANNs) consisting of massively parallel connections between artificial neurons and synapses.^[^ ^7^, ^8^, ^9^, ^10^, ^11^ ^]^ Unlike serial computing based on the conventional von Neumann architecture, HW‐ANN‐based parallel computing effectively handles unstructured data without bottleneck issues owing to the unique capability of artificial synapses that process and memorize data simultaneously.^[^ ^12^, ^13^, ^14^ ^]^ In recent years, for the implementation of HW‐ANNs, numerous studies on artificial synapses with various structures have been reported.^[^ ^15^, ^16^, ^17^, ^18^ ^]^ Early research on artificial synapses has focused mostly on two‐terminal nonvolatile memory devices based on a crossbar array structure, such as resistive random access memory, conductive bridge random access memory, and phase‐change memory.^[^ ^4^, ^19^, ^20^, ^21^, ^22^, ^23^ ^]^ This is because the crossbar array can be simply fabricated, easily expanded, and highly integrated. However, two‐terminal synaptic devices have one conducting path between the top and bottom electrodes, which is shared for writing and reading a synaptic weight. This makes it difficult to maintain synaptic weights during the read‐out process, consequently interfering with the precise weight update.^[^ ^24^ ^]^ Moreover, the two‐terminal synapse array requires specific programming schemes, such as updating with current signals and using weight‐resetting procedures to achieve the desired weight update linearity. These schemes cannot be applied for parallel computing; therefore, the two‐terminal array exploits the element‐by‐element serial weight update, which is inefficient in terms of time and energy.^[^ ^1^, ^3^ ^]^

Moreover, a three‐terminal artificial synapse based on transistor operation has been suggested owing to its structural feature enabling nondestructive read‐out and parallel‐access for updating synaptic weights. The updated weight levels are stable for a long period, and each synaptic weight can be addressed in parallel during the weight update operation. Seo et al. reported a three‐terminal synaptic device based on 2D van der Waals (vdW) materials, including hexagonal boron nitride (*h*‐BN) and tungsten diselenide (WSe_2_), where the synaptic device presented a linear weight update trajectory while providing a large number of stable conduction states with less than 1% variation per state.^[^ ^25^ ^]^ Oh et al. successfully emulated the long‐term plasticity of biological synapses with a three‐terminal synapse fabricated using vdW and self‐assembled (SA) materials with extremely small amounts of surface defects. The artificial vdW–SA synapse showed extremely stable long‐term potentiation/depression (LTP/D) characteristics with relative standard deviations (RSDs) below 2%.^[^ ^17^ ^]^ Qian et al. also proposed an organic double heterojunction to enable a nonvolatile step modulation of the conductance in a three‐terminal synapse, where the double heterojunction was consisted of *N*,*N*‐dioctyl‐3,4,9,10‐perylene tetracarboxylic diimide (PTCDI‐C_8_), copper phthalocyanine (CuPc), and *para*‐sexiphenyl (*p*‐6P). This organic synapse showed an excellent weight update characteristic with a nonlinearity (NL) below 0.01, in the LTP region and great controllability of the synaptic weight with nondestructive read‐out.^[^ ^26^ ^]^ Despite this technical progress, this three‐terminal device is less competitive than the two‐terminal device in achieving a high device density. Recently, a vertical transistor structure arrayed in a cross‐bar array structure was reported by several research groups, which seems to improve synaptic device density. Shim et al. proposed a vertical graphene‐transition metal dichalcogenide (TMD) heterojunction (GTH) transistor, where the injection from the source (graphene) to the channel (TMD) was controlled by adjusting the Fermi level (*E*_F_) of graphene.^[^ ^27^ ^]^ In this device, the active vertical channel area is a crossed region between the top and bottom electrodes, reducing the device occupying space effectively and consequently achieving a density similar to that of two‐terminal devices. In addition, Lenz et al. reported a nanoscopic vertical organic transistor operated by electrolyte gating, which showed a short channel length of less than 50 nm and nanoscopic device area of 2 × 80 × 80 nm^2^.^[^ ^28^ ^]^ More recently, Choi et al. introduced a vertical organic synapse, in which the vertical channel conductance was effectively modulated by the side ion‐gel gating effect, demonstrating its extensibility to a crossbar array structure.^[^ ^29^ ^]^ These studies showed the significant potential of the crossbar array while retaining the electrical characteristics of the three‐terminal device, but only a few studies have been reported on the application of such a structure to synaptic devices.

In this study, we developed an electrolyte‐gated vertical synapse array based on a vdW heterostructure. The synaptic weight of this synapse array preserves stably for a long period during the read‐out operation, and each synaptic device element is accessible in parallel during the weight update operation. In the vertical synapse, the long‐term plasticity of a biological synapse is successfully emulated via Fermi‐level modulation of the graphene by ion movement inside the ion‐gel weight control layer (WCL). Through electrical measurements, we first investigate the basic characteristics of an artificial synapse, such as excitatory/inhibitory postsynaptic current (E/IPSC), paired‐pulse facilitation (PPF), and LTP/D. Then, the controllability of the LTP/D characteristics, including dynamic range (*G*_max_/*G*_min_), asymmetry (AS), and effective number of conductance states (NS_eff_) are discussed, and the optimal condition of the weight control voltage (*V*_WC_) pulse is analyzed with respect to the pulse frequency, duration, and amplitude. We also evaluate the durability of the device under electrical stress and the accessibility of the conductance state under randomly repeated voltage pulses. Finally, to confirm the feasibility of the vertical synapse device array for HW‐ANNs, we theoretically perform training/inference tasks for acoustic and emotional patterns and the Modified National Institute of Standards and Technology (MNIST) digit patterns, and experimentally conduct real‐time parallel computing for training AND/OR logic functions in a small‐scale network.

## 2 Results and Discussion

### 2.1 Electrolyte‐Gated Vertical Synapse Array based on Graphene/WS_2_ Heterostructure

**Figure 1:**
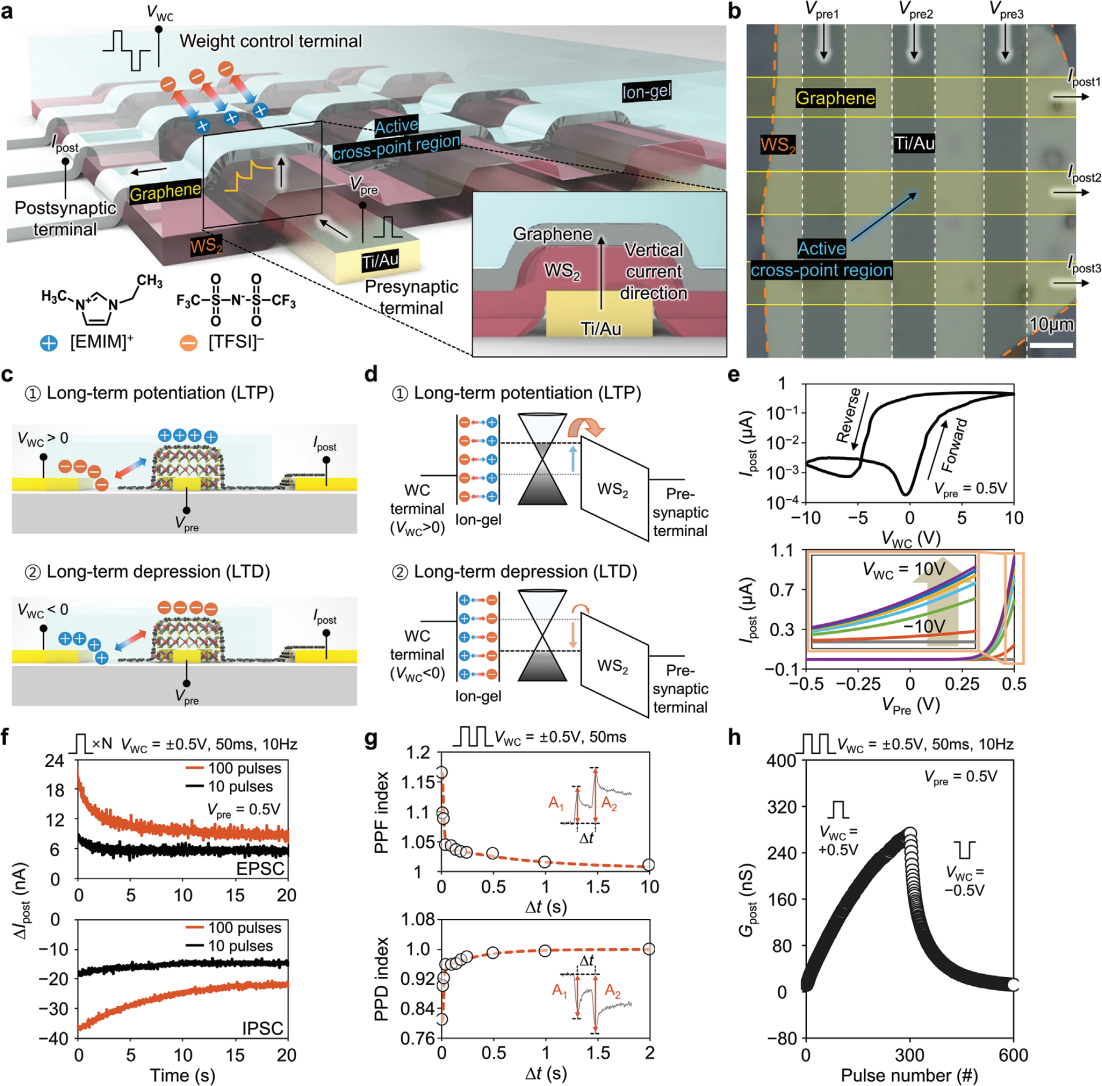
Electrolyte‐gated vertical synapse array based on graphene/WS_2_ heterostructure. a) Schematic illustration of vertical synapse array. b) OM image of vertical synapse array. c,d) Schematics and energy band diagrams showing weight updating mechanism of LTP/D. e) Current–voltage (*I*_post_–*V*_WC_ and *I*_post_–*V*_pre_) characteristics of the vertical synaptic device. f) E/IPSC responses for 20 s after applying 10 and 100 potentiating/depressing pulses. g) PPF/PPD characteristic of the vertical synaptic device. h) LTP/D characteristics of the vertical synaptic device by continuous 300 potentiating pulses (*V*_WC_ = +0.5 V, 50 ms, 10 Hz) and 300 depressing pulses (*V*_WC_ = −0.5 V, 50 ms, 10 Hz).

A synaptic cleft existing between the presynaptic and postsynaptic terminals transmits a presynaptic pulse signal generated from the presynaptic neuron to the postsynaptic neuron by releasing the neurotransmitter, and simultaneously modulates its in‐between connection strength (called “synaptic weight”). To implement such synaptic functions, we propose a vertical transistor‐type artificial synapse consisting of a graphene/tungsten disulfide (WS_2_) heterostructure and an ion‐gel. **Figure** **1**a shows the schematic images of the fabricated artificial synapse. The WS_2_ vertical channel was sandwiched between the bottom Au drain and the top graphene source electrodes, and the ion‐gel WCL covered the entire graphene electrode. The WS_2_ flake was formed on the Au bottom electrode via a precise residue‐free dry transfer technique, and the graphene top electrode was arranged on the WS_2_ flake to intersect the bottom electrode through conventional wet transfer and photolithography processes. Subsequently, the ion‐gel WCL was formed via spin coating and patterning with UV light. The main current flows via the vertical channel from the bottom to the top electrodes, where the movement of the mobile ions ([EMIM]^+^ and [TFSI]^−^) in the ion‐gel induces a long‐term change in the channel conductance, resulting in LTP and LTD of the synaptic weight. As shown in the optical microscope (OM) image of Figure 1b, the synaptic device array with a size of 3 × 3 was well fabricated, and the active cross‐point channel region was confirmed to be 10 × 10 µm^2^ (Figure 1b). We measured the thickness of the WS_2_ channel at the region indicated by the black dotted line in Figure S1a in the Supporting Information and confirmed ≈31 nm of channel thickness (Figure S1b, Supporting Information) by using atomic force microscopy (AFM) analysis. The detailed mechanism of the LTP/D characteristics is shown in the schematics in Figure 1c,d and Figure S2 in the Supporting Information. When a positive *V*_WC_ is applied to the weight control (WC) terminal, the positive ions move to the graphene source electrode, and simultaneously, the negative ions are attracted to the WC terminal. This ion movement upshifts the Fermi level of graphene, thereby lowering the injection barrier from the graphene source to the WS_2_ channel, thereby increasing the injection probability. As a result, the postsynaptic current (*I*_post_) and channel conductance (*G*_post_) increase (LTP phenomenon). On the other hand, under the application of negative *V*_WC_ pulses, the positive ions are pushed to the WC terminal and the negative ions are dragged to the graphene source electrode. This causes the Fermi level of graphene to downshift, resulting in a decrease in *G*_post_ (LTD phenomenon).

The postsynaptic current–weight control voltage (*I*_post_–*V*_WC_) characteristic supports the physical mechanism of the long‐term plasticity via the movement of the ions by *V*_WC_ (the upper panel of Figure 1e). When *V*_WC_ was changed from −10 to +10 V (1st sweep, forward direction), *I*_post_ maintained its low current state and started increasing at ≈0 V. This is probably because the initially applied large negative *V*_WC_ (−10 V) attracted many negative ions to the graphene source electrode. Therefore, *I*_post_ might be limited to a low state for a long period until the negative ions are released back from the graphene. On the other hand, when reducing *V*_WC_ from +10 to −10 V (2nd sweep, reverse direction), *I*_post_ maintained its high current state approximately up to −5 V and began to decrease thereafter. This is because the large positive *V*_WC_ (+10 V) pushed many of the positive ions to the graphene region, resulting in a high conductance state for a long period. Similar trends were observed in all other devices in the array, clearly indicating that our array has low device‐to‐device variation (Figure S3, Supporting Information). This clear hysteresis loop with counterclockwise directionality is a typical characteristic of transistor‐type memory devices that exploit electrolyte gating.^[^ ^30^, ^31^, ^32^, ^33^, ^34^, ^35^, ^36^ ^]^ The postsynaptic current–presynaptic voltage (*I*_post_–*V*_pre_) characteristic also confirmed the operating mechanism of the electrolyte‐gated vertical synaptic device (the lower panel of Figure 1e). *I*_post_ was modulated by *V*_WC_ only when the positive *V*_pre_ was applied, which clearly indicates that the electrolyte‐gated modulation by *V*_WC_ only affected the injection probability from the graphene source to the WS_2_ channel. Additionally, we investigated the gate leakage current of the vertical synaptic transistor (Figure S4, Supporting Information). The leakage current was distributed from 10^−9^ to 10^−11^, which was much lower than the channel current. This also supports that the vertical channel was well‐controlled by the ion‐gel gating effect.

To evaluate the detailed synaptic characteristics, we monitored the response of *I*_post_ after applying 10 and 100 consecutive *V*_WC_ pulses of ±0.5 V, 50 ms, and 10 Hz to the WC terminal (Figure 1f). After the application of 10 positive *V*_WC_ pulses, *I*_post_ began decreasing quickly, but it did not return to its initial value (Δ*I*_post_ > 0) and maintained a certain value even after 20 s. This behavior was also observed after applying 100 positive pulses, where a larger *I*_post_ was retained after 20 s. This indicates that the vertical synaptic device exhibited an EPSC response with respect to the positive *V*_WC_ pulse numbers. On the other hand, when applying the negative *V*_WC_ pulses, *I*_post_ remained after 20 s, indicating an IPSC response. Such E/IPSC responses are crucial because they show the controllability of the conductance according to the *V*_WC_ pulses. We also investigated the PPF/PPD characteristic because a biological synapse responds according to the time interval (∆*t*) between two consecutive pulses (Figure 1g). To measure the PPF/PPD characteristics, we applied two different *V*_WC_ pulses (+0.5 V/50 ms for PPF and −0.5 V/50 ms for PPD) with ∆*t* from 0.005 to 2 s, and then extracted the PPF/PPD index by monitoring the response of *I*_post_. The PPF index is defined as the ratio of the first and second peaks (*A*_2_/*A*_1_), and the PPD index is calculated as the ratio of the second and first peaks (*A*_1_/*A*_2_). As shown in the PPF index versus ∆*t* curve (the upper panel of Figure 1g), the PPF index exponentially decreases as ∆*t* increases, which is similar to the biological synaptic response. In the PPD index versus ∆*t* curve (the lower panel of Figure 1g), the opposite trend was observed, where the PPF index exponentially increases as ∆*t* decreases. The relationship between the PPF/PPD index and the time interval can be described with a double exponential decaying/growing formulation: PPF/PPD index = 1 + *C*_1_ exp (−Δ*t*/*τ*_1_) + *C*_2_ exp(−Δ*t*/*τ*_2_)/ 1 − *C*_3_ exp (−Δ*t*/*τ*_3_) − *C*_4_ exp(−Δ*t*/*τ*_4_)/, where *C*_1_/*C*_3_ and *τ*_1_/*τ*_3_ denote initial facilitation/depression magnitude and relaxation time in rapid phase, respectively, and *C*_2_/*C*_4_ and *τ*_2_/*τ*_4_ indicate the values in slow phase. Our synaptic device showed *τ*_1_/*τ*_3_ of 13.6/9.58 ms and *τ*_2_/*τ*_4_ of 836.12/328.95 ms for PPF/PPD, which are similar to those of a biological synapse.^[^ ^29^, ^37^, ^38^, ^39^ ^]^ We then confirmed the LTP/D characteristics, which are important synaptic characteristics that strongly influence the system‐level performance of HW‐ANNs (Figure 1h). When we applied continuous 300 potentiating and 300 depressing pulses (*V*_WC_ = ±0.5 V, 50 ms, and 10 Hz) to the WC terminal, *G*_post_ increased almost linearly and decreased completely to the initial value. This gradual conductance modulation shown in the LTP/D characteristic curve shows that the device possesses a multilevel conductance state between the maximum (*G*_max_) and minimum (*G*_min_) conductance values.

### 2.2 Controllability of LTP/D Characteristics in Electrolyte‐Gated Vertical Synaptic Device

**Figure 2:**
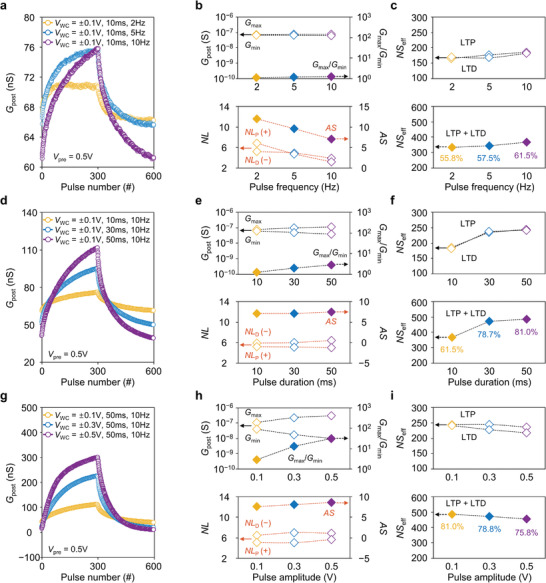
Controllability of LTP/D characteristics in electrolyte‐gated vertical synaptic device. a) LTP/D characteristic curves under various pulse frequency (2, 5, and 10 Hz). b,c) Extracted *G*_max_/*G*_min_, AS, and NS_eff_ values with respect to the pulse frequencies. d) LTP/D characteristic curves under various pulse duration (10, 30, and 50 ms). e,f) Extracted *G*_max_/*G*_min_, AS, and NS_eff_ values with respect to the pulse durations. g) LTP/D characteristic curves under various pulse amplitude (±0.1, ±0.3, ±0.5 V). h,i) Extracted *G*_max_/*G*_min_, AS, and NS_eff_ values with respect to the pulse amplitudes.

Among the various factors determining the LTP/D characteristics, *G*_max_/*G*_min_, AS, and NS_eff_ are known to be some of the most important factors that directly influence the system performance of HW‐ANNs.^[^ ^24^, ^40^, ^41^, ^42^ ^]^ The desired value of each factor depends on the structure and algorithm of HW‐ANNs, but it is generally known to require high *G*_max_/*G*_min_, low AS, and large NS_eff_ to achieve high‐performance neural networks.^[^ ^24^, ^40^, ^41^, ^43^ ^]^ Therefore, it is necessary to investigate the controllability of the LTP/D characteristics in artificial synapses and to determine the optimal *V*_WC_ pulse condition for obtaining desirable values. Here, *G*_max_/*G*_min_ was calculated as the ratio between *G*_max_ and *G*_min_, and AS was defined as the difference between the nonlinearities of the LTP and LTD regions (NL_P_ − NL_D_) (see the Experimental Section for the detailed equations of the nonlinearity calculation). NS_eff_ was obtained as the number of conductance states when the ratio of Δ*G* to *G*_max_−*G*_min_ was greater than 0.05%. To evaluate the LTP/D characteristics of the vertical synaptic device, we monitored the response of *G*_post_ while applying continuous 300 potentiating and 300 depressing *V*_WC_ pulses under various pulse frequencies, durations, and amplitudes. *V*_pre_ was fixed as 0.5 V during all measurements in **Figure** **2**. As the pulse frequency increased from 2 to 10 Hz, *G*_max_ increased slightly from 71.1 to 75.8 ns and *G*_min_ decreased slightly from 66 to 61 ns, as shown in Figure 2a and the upper panel of Figure 2b, where the amplitude and duration of the pulses were fixed at ±0.1 V and 10 ms, respectively. As a result, *G*_max_/*G*_min_ barely changed from 1.08 to 1.24, which were lower than the desired values (*G*_max_/*G*_min_ > 10) for achieving high‐performance neural networks.^[^ ^24^ ^]^ In contrast, both NL_P_ and NL_D_ decreased significantly as the pulse frequency increased, resulting in a remarkable decrease in AS from 11.99 to 7.06 (the lower panel of Figure 2b). In addition, owing to the considerable improvement in the linearity and the constant dynamic range, NS_eff_ of the LTP and LTD regions increased from 165 to 186 and from 170 to 183, respectively (upper panel of Figure 2c). As a result, the total NS_eff_ increased from 335 to 369, indicating that the ratio of the usable conductance state to the total of 600 states increased from 55.8% to 61.5%, as shown in the lower panel of Figure 2c. We also investigated the higher frequency response of *G*_post_ in the synaptic device (Figure S5, Supporting Information). Our vertical synapses exhibited gradual conductance modulation in the LTP/D curves even at a pulse frequency of 100 Hz, where *G*_max_/*G*_min_ = 7.5, AS = 4.9, and NS_eff_ = 709 were confirmed after the application of 500 consecutive pulses. We then analyzed *G*_post_ by applying *V*_WC_ with various pulse durations of 10, 30, and 50 ms, where the amplitude and frequency were fixed at ±0.1 and 10 Hz, respectively (Figure 2d). As the pulse duration increased from 10 to 50 ms, *G*_max_/*G*_min_ increased from 1.24 to 2.85 (top panel of Figure 2e). This is probably because the energy delivered by a single *V*_WC_ pulse increases as the pulse duration increases. Meanwhile, AS was not affected by the pulse duration change and was maintained near 7 (bottom panel of Figure 2e). Owing to the enhancement of *G*_max_/*G*_min_ and the constant AS value, the total NS_eff_ increased from 369 to 486; in particular, in the 50 ms pulse duration condition, 81.0% of the total conductance states were usable (Figure 2f). We fixed the pulse frequency and duration at 10 Hz and 50 ms, respectively, and investigated the effect of the pulse amplitude on the *G*_post_ response (Figure 2g). As the pulse amplitude increased from ±0.1 to ±0.5 V, *G*_max_/*G*_min_ increased significantly from 2.85 to 31.3 with a slight degradation of AS from 7.52 to 8.56. It seems that these values are sufficient to achieve high training accuracy in our designed HW‐ANNs. NS_eff_ decreased slightly from 486 to 455, but 75.8% of the total conductance states could still be available. Additionally, we calculated the read (*E*_read_) and write (*E*_write_) energy consumption for the LTP/D characteristics of the vertical synaptic device (Figure S6, Supporting Information). Through the evaluation and optimization processes, we confirmed that our synaptic device possessed a sufficiently large dynamic range (*G*_max_/*G*_min_ = 31.3), weight update linearity (AS = 8.56), and a sufficient number of conductance states between *G*_max_ and *G*_min_ (NS_eff_ = 75.8%). In addition, we compared the nonlinearity and asymmetry of LTP/LTD characteristics of our synaptic device with the values of state‐of‐the‐art devices in Table S1 in the Supporting Information, and we then confirmed that our values were slightly worse than the others. Such high nonlinearity and asymmetry values can be improved by several schemes, which include i) pulse optimization^[^ ^26^, ^44^ ^]^ and ii) synaptic unit cell design.^[^ ^45^, ^46^ ^]^

### 2.3 Stability of Electrical Performance in Electrolyte‐Gated Vertical Synaptic Device

**Figure 3:**
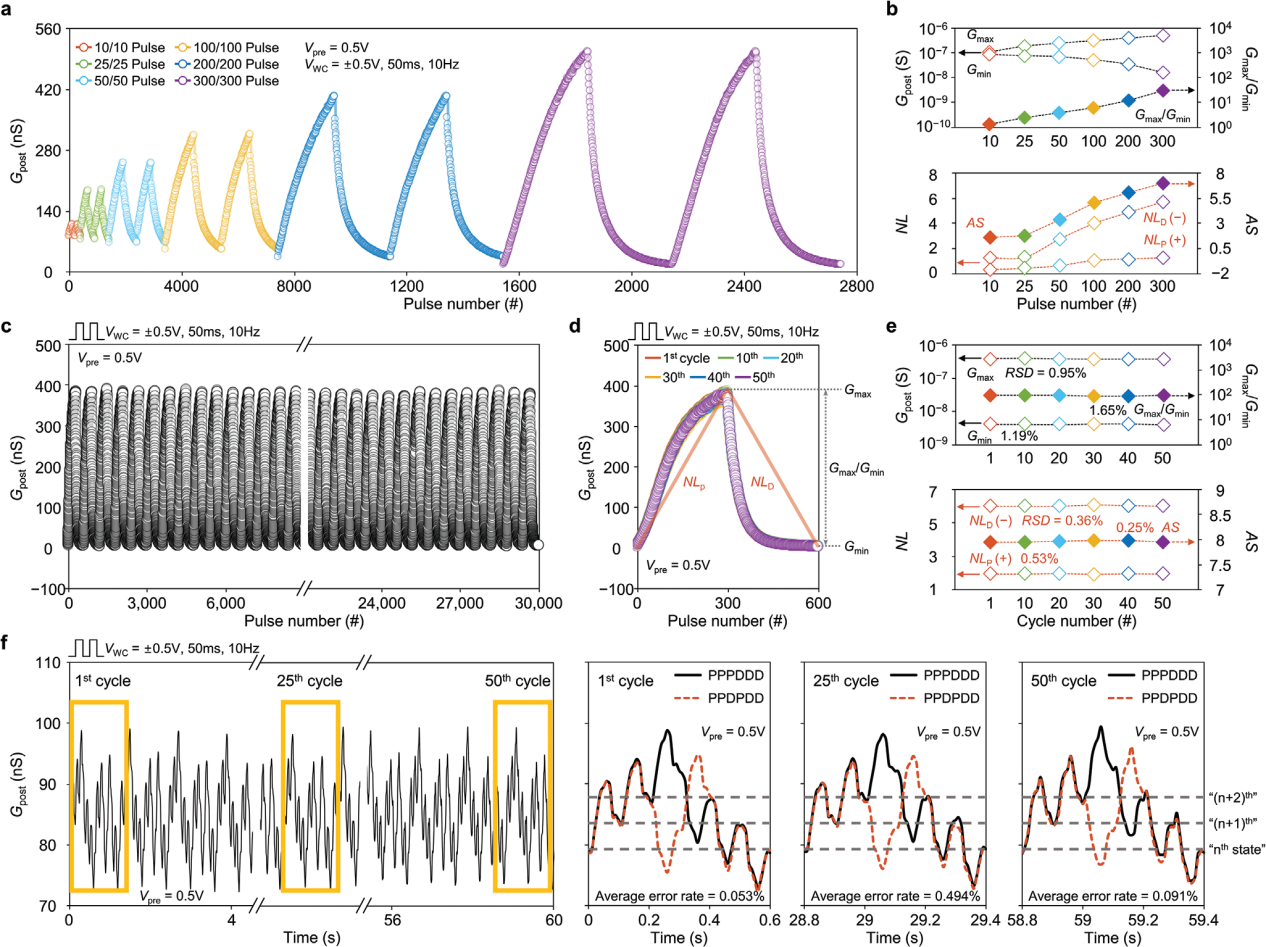
Stability of electrical performance in electrolyte‐gated vertical synaptic device. a) LTP/D characteristic curves of the vertical synaptic device under different number of potentiation/depression pulses, ranging from 10/10 to 300/300. b) Extracted *G*_max_/*G*_min_ and AS values for the numbers of potentiation/depression pulses. c) LTP/D characteristic curves of the vertical synaptic device over 50 cycles, where one cycle consisted of 300 potentiating and 300 depressing pulses. The amplitude, duration, and frequency of *V*_WC_ pulse were ±0.5 V, 50 ms, and 10 Hz. d) LTP/D characteristic curves of 1st cycle and every 10th cycle from 10th to 50th. e) Extracted *G*_max_/*G*_min_ and AS values for 50 LTP/D cycles. f) State stability under two different sequential pulse sets (“PPPDDD” and “PPDPDD”) and *G*_post_ plots in 1st, 25th, and 50th cycles.

For the reliable operation of HW‐ANNs consisting of electronic synaptic devices, synaptic performance stability against electrical stress should also be secured.^[^ ^40^, ^43^ ^]^ **Figure** **3**a shows the LTP/D characteristic curves over two cycles with respect to a different number of pulses, ranging from 10 to 300, where *V*_WC_ was fixed at ±0.5 V, 50 ms, and 10 Hz. The LTP/D curves for all cases were similar in shape; *G*_post_ increased gradually with the potentiating pulses and returned to the initial value by the depressing pulses. This shows that our synaptic device can maintain its gradual conductance modulation characteristics regardless of the number of pulses. The *G*_max_/*G*_min_ and AS values increased gradually from 1.33 to 30.62 and from 1.56 to 6.96, respectively, showing the trade‐off relationship between the dynamic range and linearity with respect to the number of pulses (Figure 3b).^[^ ^29^, ^30^ ^]^ To investigate the device endurance specifically, we monitored the LTP/D characteristic curves over 50 cycles corresponding to 30 000 pulses while applying consecutive 300 potentiating and 300 depressing pulses to the WC terminal for a cycle. As shown in Figure 3c, our synaptic device presented highly robust LTP/D characteristics against electrical stress, where the dynamic conductance modulation range was maintained over the entire test cycle. We then plotted the LTP/D curves of the 1st and 10th cycles from 10th to 50th (Figure 3d) and confirmed that the six LTP/D curves were highly similar to each other in shape. To evaluate the endurance quantitatively, we extracted *G*_max_/*G*_min_ and AS for each LTP/D characteristic curve and calculated the RSD for the six curves (Figure 3e). For all test cycles, the *G*_max_ and *G*_min_ values were maintained, and thus, the RSD for *G*_max_/*G*_min_ was only 1.65%. In addition, NL_P_ and NL_D_ remained unchanged even after 50 cycles; in particular, the RSD of AS was extremely low at 0.25%. This result indicates that our synaptic device is highly stable in terms of its dynamic characteristics against electrical stress. We also investigated the *G*_post_ response to the two different sequential pulse sets of “PPPDDD” (black solid line) and “PPDPDD” (red dotted line) for 50 set cycles, where P and D denote potentiating and depressing pulses, respectively (Figure 3f). In the 1st cycle, each conductance value corresponding to the *n*th, (*n*+1)th, and (*n*+2)th states of the pulse set “PPPDDD” perfectly matched to that of the pulse set “PPDPDD.” The error rate of each conductance state was only 0.005%, 0.128%, and 0.025%, which indicates that our synaptic device has highly stable conductance states that can be accessible at any time. This excellent state stability was observed even after the 25th and 50th cycles, and their average error rates over the three states were extremely low at 0.494% and 0.091%, respectively.

### 2.4 Training and Inference Tasks for Acoustic and Emotional Patterns

**Figure 4:**
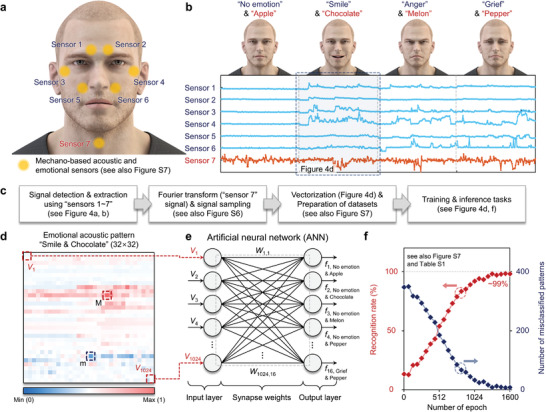
Training and inference tasks for acoustic and emotional patterns. a) Schematic illustration of mechano‐based acoustic and emotional sensors attached onto the forehead, eyes, nose, and neck. b) Extracted sensor signals consisting of four acoustic signals and four emotional signals. c) Training and inference tasks for the acoustic and emotional patterns. d) 32 × 32 mapping image of “Smile & Chocolate,” where synaptic weights were distributed between 0 from 1. e) Single‐layer ANN consisting of input neuron layer, synapse layer, and output neuron layer. f) Recognition rate and number of misclassified patterns among acoustic and emotional patterns.

Finally, we designed a novel sensory‐neuromorphic system for acoustic and emotional information translation, where the system was consisted of carbon nanotube (CNT)/graphene/SEBS (polystyrene‐*block*‐poly(ethylene‐ran‐butylene)‐*block*‐polystyrene) sensors for detecting acoustic and emotional signals and a vertical synaptic device array for interpreting the patterned data. Then, the applicability of the vertical synaptic devices to the designed system was confirmed via an artificial neural network (ANN) simulation using acoustic and emotional patterns obtained by the mechano‐based voice and face motion sensors. Because voice and face motion signals are critical information for humans to express their condition, the human brain receives such visual and aural information and judges the condition of others. As the first step to mimic this brain function with the ANN consisting of our electrolyte‐gated vertical synapse array, we measured voice and face motion signals simultaneously and then transformed the signals into acoustic and emotional patterns. The process is as follows: i) attaching several mechano‐based acoustic and emotional sensors to the forehead, eyes, nose, and neck (**Figure** **4**a and Figure S7a, Supporting Information) and measuring signals related to voice and face motion (Figure 4b), ii) sampling the amplitude components of the voice and face motion signals as a function of time, and additionally, transform the time‐domain information to the frequency domain by a Fourier transform to obtain the width components of the signals (Figure S7b, Supporting Information), and iii) converting the discrete signal information to acoustic and emotional mapping images with a 32 × 32 array size (Figure 4d and Figure S7 in the Supporting Information for acoustic and emotional mapping images, and Figure S7d in the Supporting Information for pattern recognition rate with respect to the array size). We then designed a single‐layer ANN consisting of 1024 input neurons, 16 output neurons, and 1024 × 16 synapses, where the characteristics of 1024 × 16 synapses were calibrated with experimentally obtained synaptic characteristics such as the nonlinearity of LTP/LTD characteristics, the *G*_max_ value of LTP/LTD characteristics, the *G*_max_/*G*_min_ ratio, and the number of conductance states (Tables S1 and S2, Supporting Information). Following the design of the single‐layer ANN, the training and inference tasks for combined patterns were performed, in which the patterns include “Apple,” “Chocolate,” “Melon,” and “Pepper” acoustic data and “No emotion,” “Smile,” “Anger,” and “Grief” emotional data (Figure 4e and Figure S8, Supporting Information). The voltage signals (*V_n_*), which correspond to the pixels in the acoustic and emotional mapping images, were assumed to be applied to the input neurons. They were then multiplied with the synaptic weights and summed at the output neurons $(I_{m}=\sum_{n=1}^{1024}W_{n,m}V_{n})\;$, where synapse weights are related to the conductance (*G*) values extracted from our vertical synaptic device. The synaptic weight is defined as the conductance difference between two equivalent vertical synaptic devices (*W* = *G*_P_ − *G*_D_). This is because the conductance values in HW‐ANNs are normally positive so that it is difficult to express the inhibitory synaptic connection. In this light, the conductance difference representation between two equivalent devices can be one of the solutions to achieve both excitatory and inhibitory synaptic weights.^[^ ^17^, ^45^, ^47^ ^]^ Next, the output values (*f_m_*) were calculated using the sigmoid activation function $\left(f\left(I_{m}\right)=\frac{1}{1+e^{-I_{m}}}\right)$ and compared with the corresponding label values (*k_m_*). Finally, if necessary, the weight values were updated using the backpropagation algorithm (details are presented in the Experimental Section). This training process was repeated with 1600 sets of training data, and the recognition rate was estimated with 400 sets of testing data every training epoch. 1600 sets of training data and 400 sets of testing data were achieved via repeated measurements. Figure S9a in the Supporting Information shows the weight matrices after training partially (10th epoch) and completely (1600th epoch) the “Chocolate” acoustic and “Smile” emotional information, where synaptic weights were distributed in the range from 0 to 1. After the 10th training step, the synaptic weight map still showed a random distribution of values between 0 and 1 (the left panel of Figure S9a, Supporting Information). However, some synaptic weights were intensively updated after the 1600th training step (the right panel of Figure S9a, Supporting Information). The confusion matrix, actual (input) versus predicted (output), also shows that the constructed ANN was successfully trained after the 1600th training step (Figure S9b, Supporting Information). We then plotted the recognition rates for the acoustic and emotional information patterns as a function of the training epoch. As shown in Figure 4f, our synaptic device, in which the LTP/D characteristics were optimized, assisted the ANN to reach an ≈99% of maximum recognition rate. Additionally, we confirmed the applicability of our synaptic devices for HW‐ANNs in two other ways (Figure S10, Supporting Information): i) real‐time parallel computing for training AND/OR logic gate functions in a small‐scale network^[^ ^29^, ^48^, ^49^ ^]^ and ii) training and inference tasks for MNIST digit patterns using the *NeuroSim*+ MLP simulator.^[^ ^40^ ^]^ These results show that our synaptic devices can be used to implement parallel computing networks.

## 3 Conclusion

In this study, we realized an electrolyte‐gated vertical synapse array using a WS_2_/graphene heterojunction and an ion‐gel layer. The long‐term plasticity, which is one of the important characteristics of biological synapses, was successfully imitated via Fermi level modulation by ion movement inside the ion‐gel WCL. In particular, we achieved a dynamic range (*G*_max_/*G*_min_ = 31.3) and weight update linearity (AS = 8.56) by optimizing the LTP/D characteristics under various *V*_WC_ pulse conditions. In addition, this synaptic device was highly stable, where the RSDs of *G*_max_/*G*_min_ and AS were 1.65% and 0.25%, respectively, under 30 000 sets of *V*_WC_ pulses consisting of 300 potentiating pulses and 300 depressing pulses. Finally, we verified the feasibility of our vertical synapse array for parallel computing networks through the training and inference tasks for acoustic and emotional patterns using a single layer ANN with a size of 1024 × 16, and a maximum recognition rate of ≈99% was achieved using the optimized LTP/D characteristics. Research on vertical synaptic devices and their application to networks is expected to provide essential and fundamental information for the implementation of future parallel computing networks.

## 4 Experimental Section

### Fabrication of Vertical Synaptic Device

The electrode for the postsynaptic terminal was patterned on a heavily p‐doped silicon substrate with a thermally grown 90 nm thick SiO_2_ using a photolithography process, followed by 5 nm thick Ti and 15 nm thick Au deposition using electron‐beam evaporator. The WS_2_ flake was mechanically transferred on the patterned electrode via a residue‐free transfer method based on adhesion energy engineering.^[25]^ Subsequently, a monolayer graphene grown on Cu foil by the chemical vapor deposition method was transferred on the top of the WS_2_ flake using a wet‐transfer method to form the presynaptic terminal. The transferred graphene was patterned with a perpendicular direction of the postsynaptic electrode by using photolithography and O_2_ plasma etching processes. Finally, the ion‐gel WCL was spin‐coated and patterned by exposing the target to UV light for 40 s through the shadow mask, which was connected to the WC terminal.

### Characterization of Vertical Synaptic Device

The OM images of the device were obtained using an upright metallurgical microscope (Olympus BX53M). The thickness of the WS_2_ flake was confirmed through AFM analysis (NX10, Park Systems Corp.). Electrical characteristics of the vertical synaptic devices were monitored using a Keysight B2912A parameter analyzer. A constant voltage of 0.5 V was applied to the postsynaptic terminal to read the *I*_post_ flowing through the vertical WS_2_ channel between the presynaptic (bottom) and postsynaptic (top) terminals. For the application of the *V*_WC_ pulses, a Keysight 33500B waveform generator was simultaneously exploited and connected to the WC terminal. All electrical measurements were performed at 20 °C and relative humidity of 25% (monitored by a commercial thermo‐hygrometer).

### Fabrication of CNT/Graphene/SEBS Composite Sensor

A 2 g of SEBS H1062 elastomer pallets (Asahi Kasei Company) was dissolved into 15 mL of toluene, followed by stirring with a magnetic bar over 4 h. After SEBS pallets were fully melted into toluene, 0.6 g of CNTs (US Research Nanomaterials, Inc.) and 0.2 g of graphene oxide nanoparticles (GO, US Research Nanomaterials, Inc.) were poured into the solution and stirred with a magnetic bar overnight. CNT/graphene/SEBS dispersion was then poured into a petri dish to form a fine sensor film and let the toluene solvent evaporated in a fume hood overnight. Details regarding the electrical characteristics of the composite sensor are provided in Figure S12 in the Supporting Information.

### Weight Update for Vertical Synaptic Device

To update the synaptic weight, delta value (*δ* = *k* − *f*) was calculated by subtracting the output value from the corresponding label value. If *δ* > 0, *G*_P_ increased and *G*_D_ decreased, resulting in the potentiation of the synaptic weight (*W*↑ = *G*_P_↑ − *G*_D_↓). In contrast, if *δ* < 0, the synaptic weight was depressed (*W*↓ = *G*_P_↓ − *G*_D_↑). If *δ* = 0, the synaptic weight was not updated. These conductance changes were determined by the following equations

(1) $$G_{n+1}=G_{n}\;+\Delta G_{P}=G_{n}+\alpha_{P}e^{-NL_{P}\frac{G_{n}-G_{\min}}{G_{\max}-G_{\min}}}\left(\Delta G>0,G↑\right)$$

(2) $$G_{n+1}=G_{n}+\Delta G_{D}=G_{n}-\alpha_{D}e^{NL_{D}\frac{G_{\max}-G_{n}}{G_{\max}-G_{\min}}}\left(\Delta G<0,G↓\right)$$

here, *G_n_*_+1_ and *G_n_* denote the synaptic weight values when the *n*+1th and *n*th pulses are applied, and the parameters *α* and NL indicate the amount of conductance change and the nonlinearity, respectively. Details regarding the estimation of the *α* and NL values are provided in Figure S13 in the Supporting Information.

## Conflict of Interest

The authors declare no conflict of interest.

## Supporting information

Supporting Information

## Data Availability

The data that support the findings of this study are available from the corresponding author upon reasonable request.
