# Supplementary material for: Electrolyte‐Gated Vertical Synapse Array based on Van Der Waals Heterostructure for Parallel Computing
Source: Adv Sci (Weinh). 2021 Dec 26;9(6):2103808. doi: 10.1002/advs.202103808 (PMC8867203; doi:10.1002/advs.202103808)
Supplement: Supplementary file 1 — Supporting Information [file ADVS-9-2103808-s001.pdf]

WILEY-VCH

ADVANCED SCIENCE
Open Access

Supporting Information

for *Adv. Sci.*, DOI: 10.1002/advs.202103808

Electrolyte-gated vertical synapse array based on van der Waals heterostructure for parallel computing

*Seyong Oh, Ju-Hee Lee, Seunghwan Seo, Hyongsuk Choo, Dongyoung Lee, Jeong-Ick Cho and Jin-Hong Park**

Supporting Information

Electrolyte-gated vertical synapse array based on van der Waals heterostructure for parallel computing

*Seyong Oh, Ju-Hee Lee, Seunghwan Seo, Hyongsuk Choo, Dongyoung Lee, Jeong-Ick Cho and Jin-Hong Park**

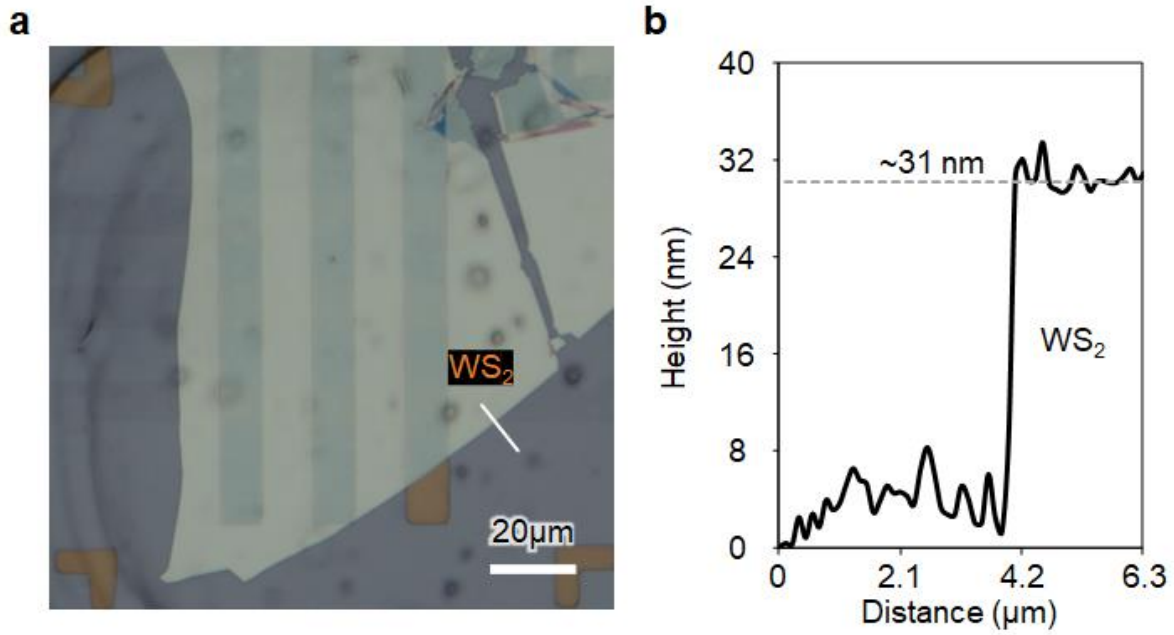

Figure S1. AFM analysis of the vertical synapse array. a) OM image of the vertical synapse array. b) Height profile of the synaptic device along the white line. The thickness of WS_2 was approximately 31 nm.

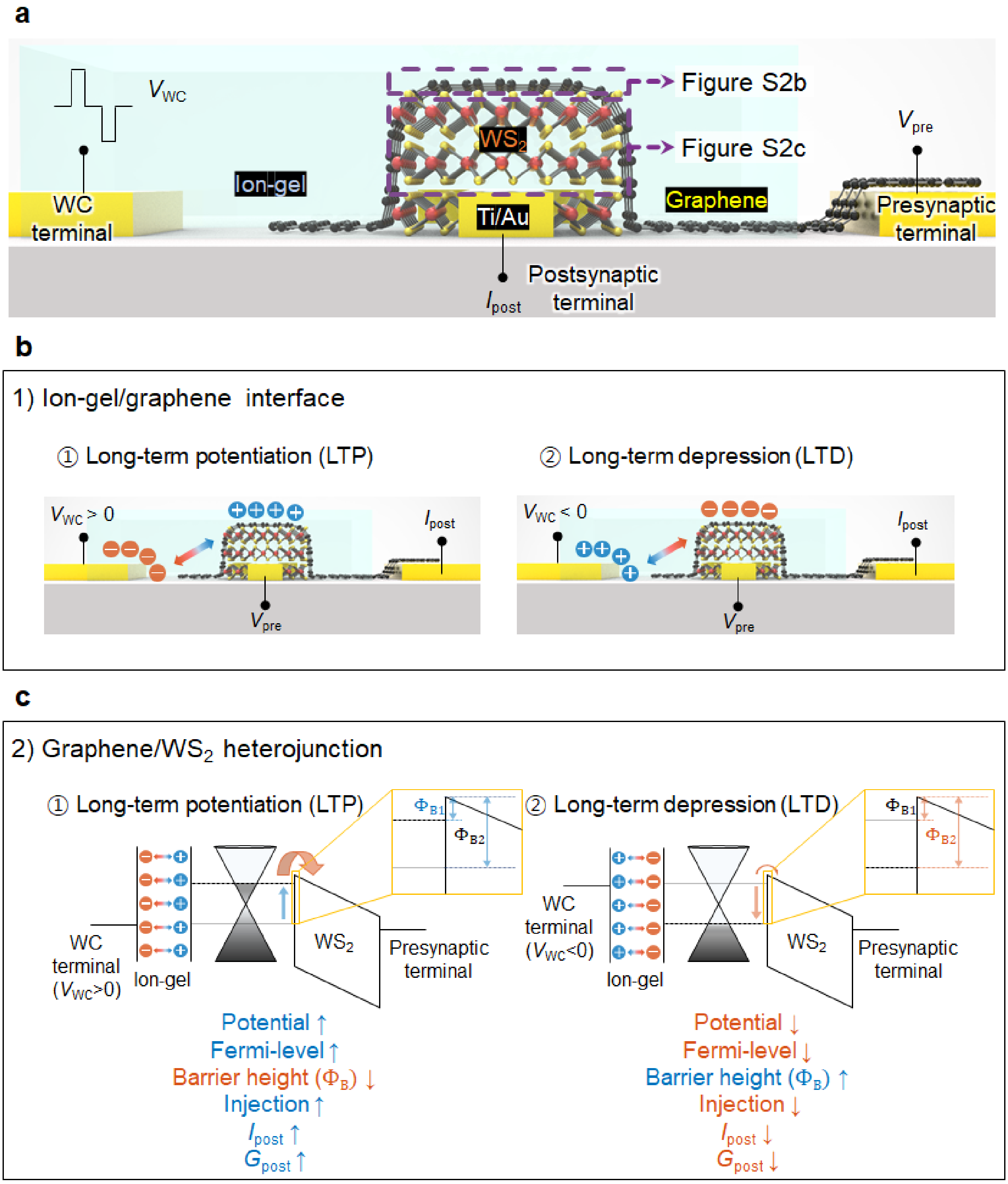

Figure S2. Weight update mechanism of the vertical synaptic device. a) Schematic illustration of the vertical synaptic device composed of the graphene/WS_2 heterostructure. b) Schematic illustration showing the ion movements at the ion-gel/graphene interface when the positive (LTP) and negative (LTD) V_{WC} pulses are applied to the WC terminal. c) Energy band diagrams of graphene and WS_2 when the positive and negative V_{WC} are applied.

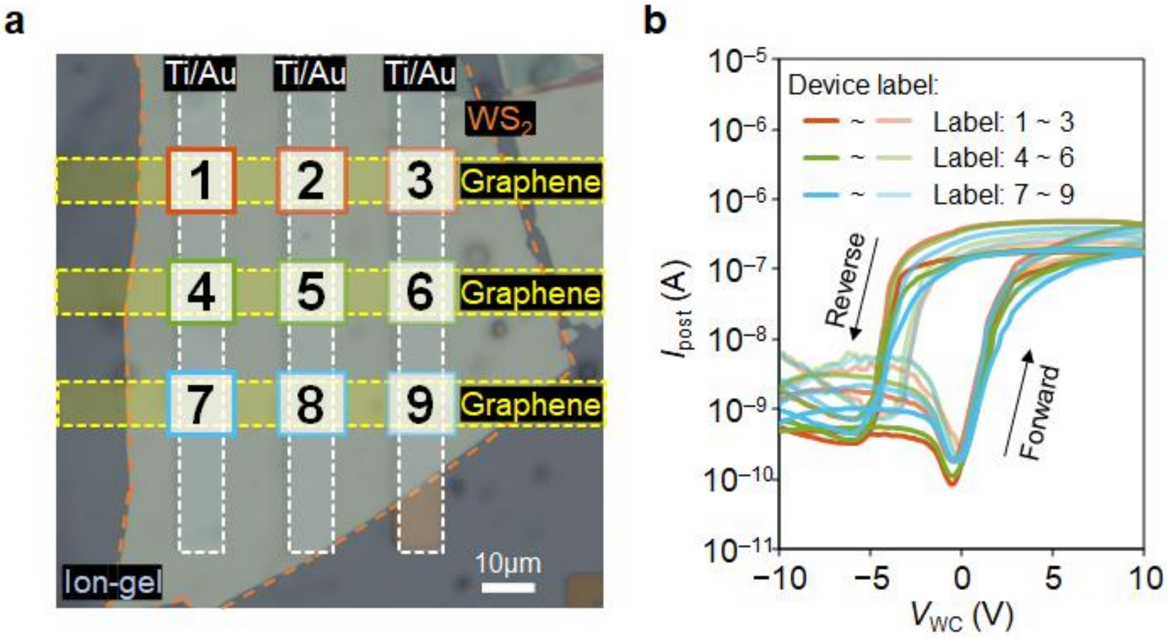

Figure S3. Device-to-device variation of the vertical synapse array. a) OM image of the vertical synapse array. b) Current-voltage (I_{post}-V_{WC}) characteristic curves of the 9 different synaptic devices in the array. The 9 devices shared the one weight control terminal in this work. Although there were slight variations in on-current and ΔV_{th}, the devices presented very similar I_{post}-V_{pre} characteristics. For the individual weight update that the reviewer was concerned about, we can suggest a strategy as follow; 1) patterning the ion-gel electrolyte as a line shape in parallel with the bottom Ti/Au electrode, and then 2) applying a compensation voltage to the graphene electrodes which should not be updated. The compensation voltage may be selected to the same as a weight control voltage to make the potential difference between the graphene and weight control electrodes zero during the updating event.

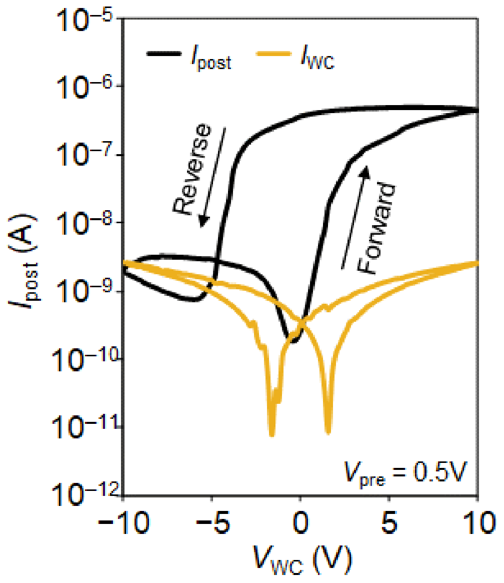

Figure S4. Current-voltage (I_{post}-V_{WC}) and gate leakage (I_{WC}-V_{WC}) characteristics of the vertical synaptic device.

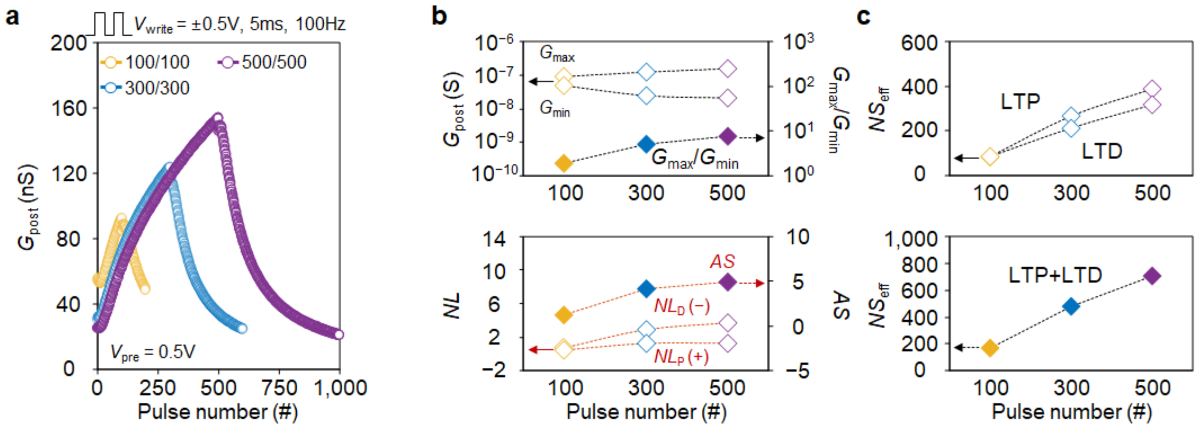

Figure S5. LTP/D characteristics at a pulse frequency of 100 Hz. a) LTP/D characteristic curves under the pulse frequency of 100 Hz, where the amplitude and duration of V_{WC} were fixed as ±0.5 V and 5 ms, respectively. b,c) Extracted G_{max}/G_{min}, *AS*, and NS_{eff} values with respect to the pulse number from 100 to 500.

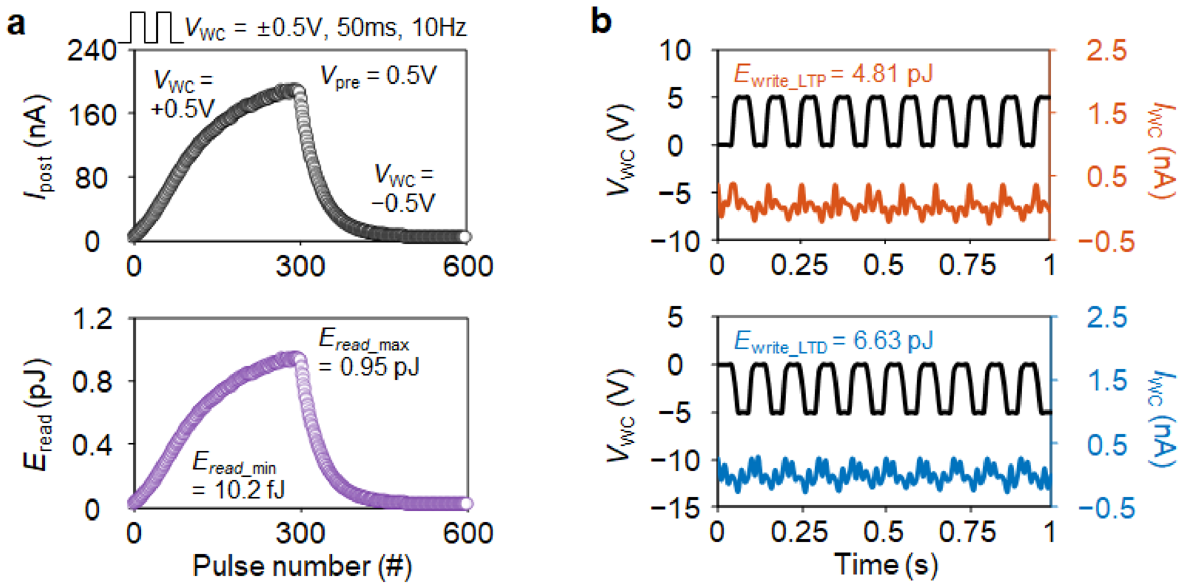

Figure S6. Energy consumption of the vertical synaptic device. a) Measured I_{post} (upper panel) and calculated E_{read} (bottom panel) curves under consecutive 300 potentiating and 300 depressing pulses. b) Measured I_{WC} and calculated E_{write_LTP} & E_{write_LTD} curves under the potentiation (upper panel) and depression pulses (bottom panel).

We approximately calculated the reading energy (E_{read}) and writing energy (E_{write}) using the equations below:

$$E_{read} = V_{pre} \times I_{post} \times t_{pre} \quad (1)$$

$$E_{write} = V_{WC} \times I_{WC} \times t_{WC} \quad (2)$$

where V_{pre}, I_{post}, and t_{pre} symbolize the magnitude of V_{pre}, the value of I_{post}, and the pulse duration of V_{pre}, respectively. V_{WC}, I_{WC}, and t_{WC} mean the magnitude of V_{WC}, the peak value of I_{WC}, and the pulse duration of V_{WC}, respectively.[S1,S2] As shown in the bottom panel of Figure S6a, E_{read} was distributed from E_{read_min} (0.5 V × 2.04 nA × 10 μs = 10.2 fJ) to E_{read_max} (0.5 V × 1.89 nA × 10 μs = 0.95 pJ). In addition, the values of E_{write_LTP} and E_{write_LTD} were calculated as 0.5 V × 0.192 nA × 50 ms = 4.81 pJ and −0.5 V × −0.265 nA × 50 ms = 6.63 pJ, respectively.

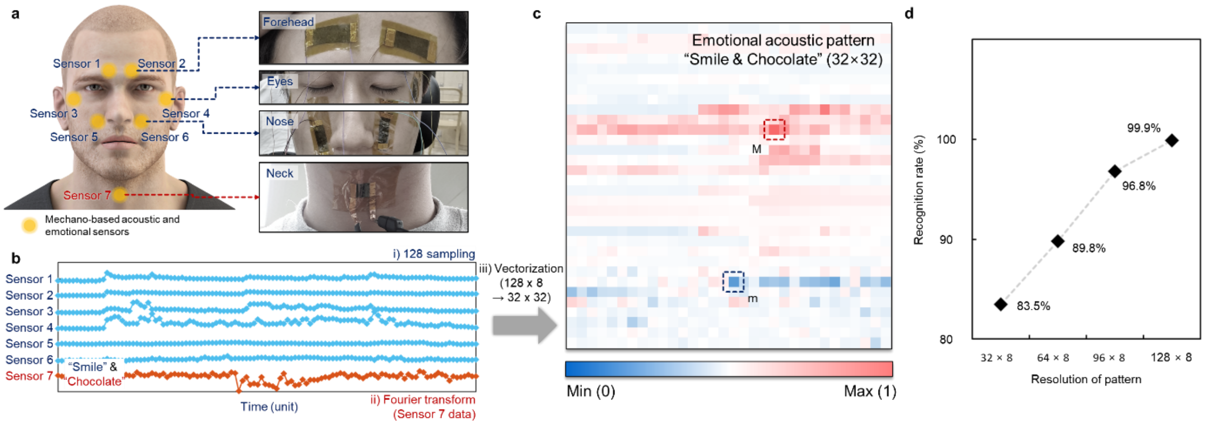

Figure S7. Voice and face motion signals extracted via mechano-based acoustic and emotional sensors. a) Schematic (left) and photographic (right) images showing the attaching position of the sensors such as the forehead, eyes, nose, and neck. b) Sampled voice and face motion signals for the "Smile & Chocolate." c) 32 × 32 synaptic weight mapping image of "Smile & Chocolate", where the synaptic weights are distributed between 0 and 1. d) Pattern recognition rate with respect to the resolution of pattern.

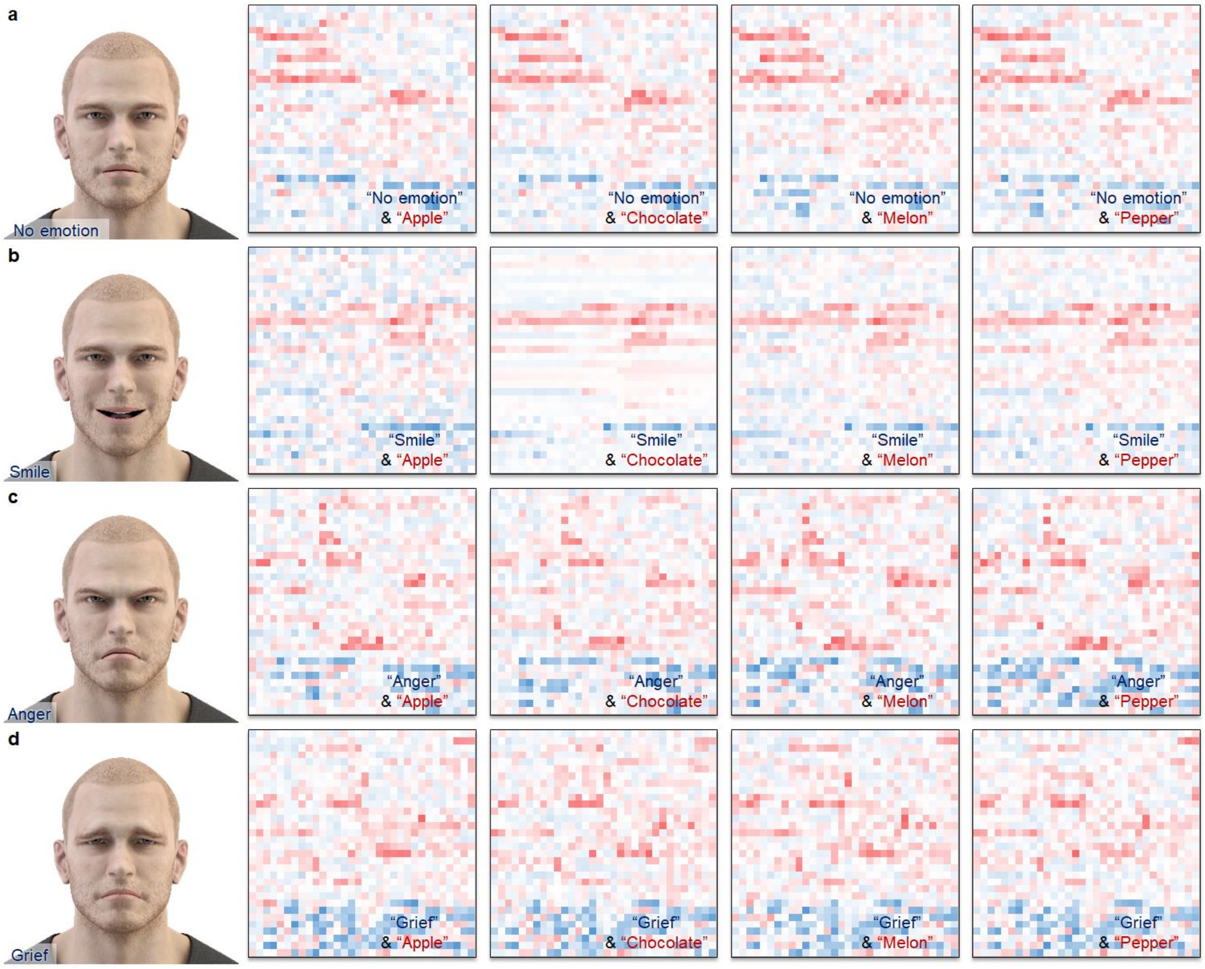

Figure S8. Combined synaptic weight mapping images of emotional patterns corresponding to "No emotion", "Smile", "Anger", and "Grief" and acoustic patterns for "Apple", "Chocolate", "Melon", and "Pepper". a,b,c,d) 16 combined acoustic and emotional signal datasets with a size of 32 × 32.

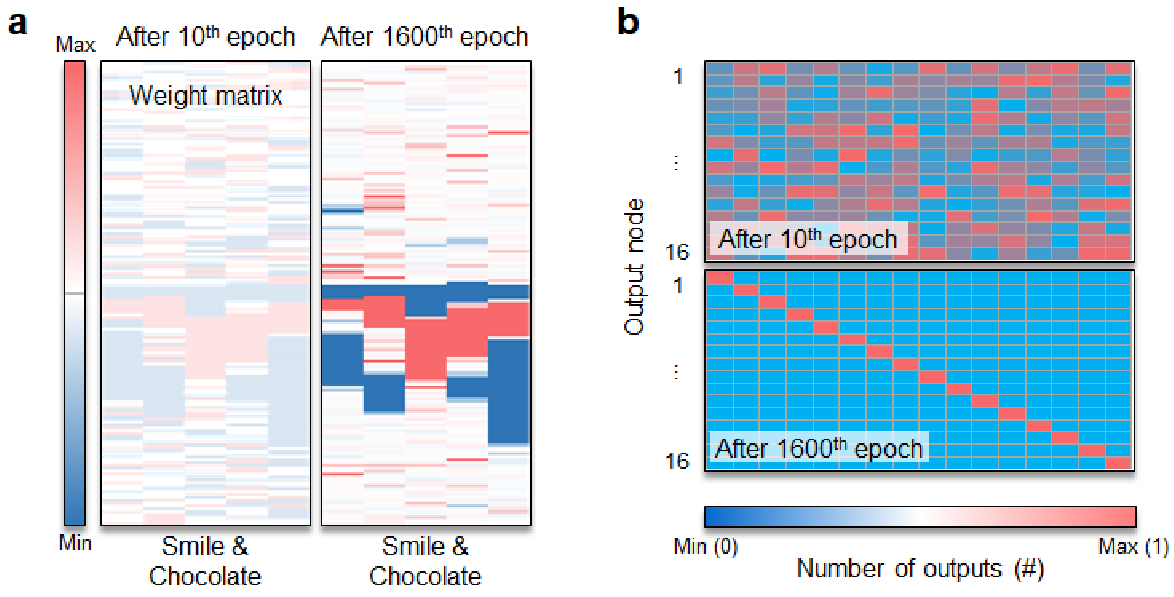

Figure S9. Comparison before and after training for acoustic and emotional information patterns. a) Synaptic weight mapping images after training 10th and 1600th epochs. b) Confusion matrix (actual (input) vs. predicted (output)) of acoustic and emotional signals after the 1600th epoch.

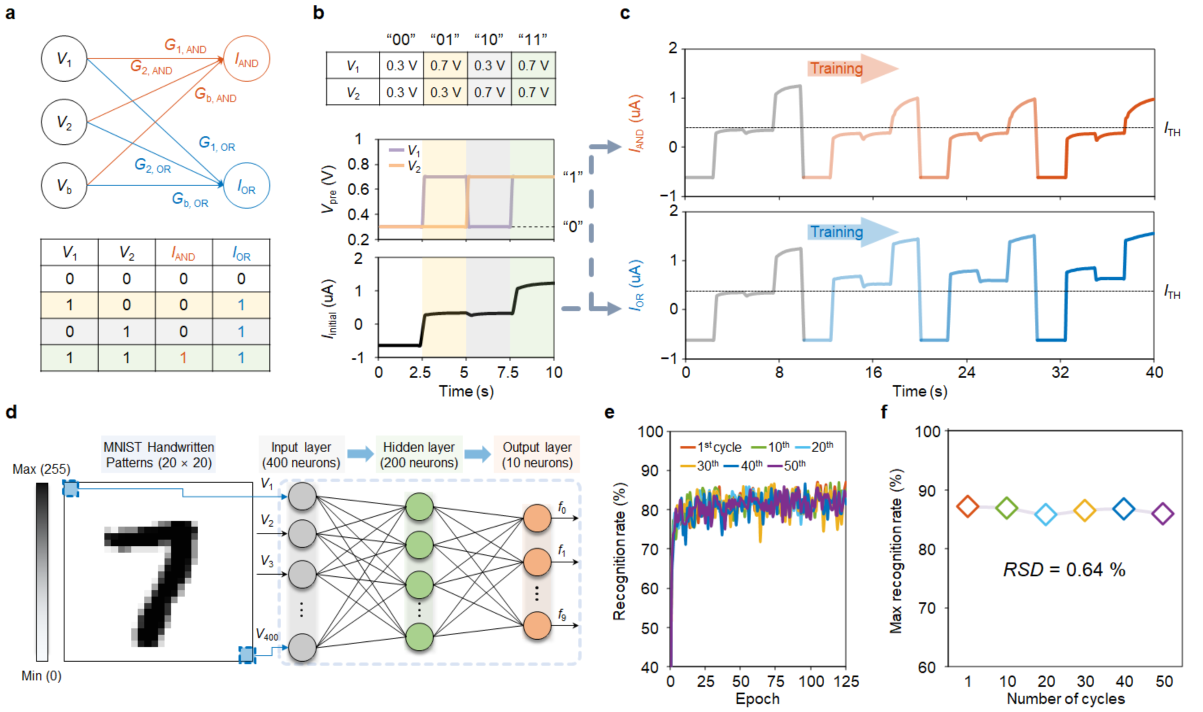

V_1	V_2	I_{AND}	I_{OR}
0	0	0	0
1	0	0	1
0	1	0	1
1	1	1	1

Figure S10. AND/OR application of vertical synapse array and training/recognition tasks. a) Schematic illustration of AND/OR logic gates using the vertical synaptic array with a size of 2 × 3 and truth table of AND/OR logic gates. b,c) Real-time training and classification of AND/OR gates. d) Schematic illustration of multi-layer perceptron with a size of 400 × 200 × 10. e,f) Recognition rates under LTP/D characteristics of the 1st and every 10th cycle from 10th to 50th and relative standard deviation (*RSD*) of maximum recognition rate of each cycle.

Small-scale neural networks of AND and OR gates are shown in Figure S10a. V_1 and V_2 denote two logic inputs, V_b denotes a bias voltage, I_{AND} and I_{OR} denote the output currents, and G means a conductance value of our synaptic device. Figure S10b shows the initial current signal when applied two logic input voltages. Two logic input voltages values of 0.3 V and 0.7 V were used for logic states of "0" and "1", respectively. If the current signal is less than 0.4 µA, the state is "0"; if not, the state is "1". As shown in Figure S10c, all conductance values of the synaptic array were updated in real-time and we analyzed output currents when the two logic inputs were "00", "10", "01", and "11", respectively. Before training, the output current signals of "10"and "01" states were close to I_{TH}, so that it was difficult to distinguish AND or OR operations. However, after 3 training times, both AND or OR operations were successfully distinguished. Furthermore, we theoretically constructed large-scale neural networks with a size of 400 × 200 × 10 with the measured LTP/D characteristics of the 1st cycle and every 10th cycle from 10th to 50th (Figure S10d). We then performed training and inference tasks for the Modified National Institute of Standards and Technology (MNIST) digit patterns using the *NeuroSim+* MLP simulator.[S3] Figures S10e and f show recognition rates with respect to various LTP/D cycles. Our synaptic device shows a maximum recognition rate of 87.19% and extremely stable cycle-to-cycle variation with *RSD* below 1%.

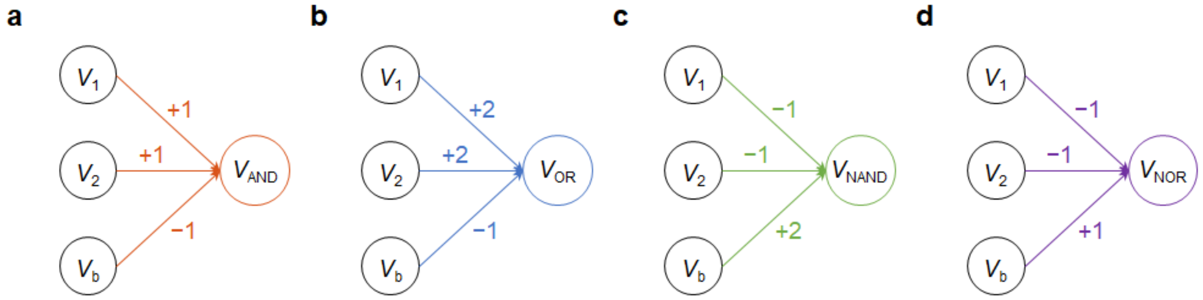

Figure S11. Schematic illustration of the neural network configurations for four logic functions such as a) AND, b) OR, c) NAND, and d) NOR.

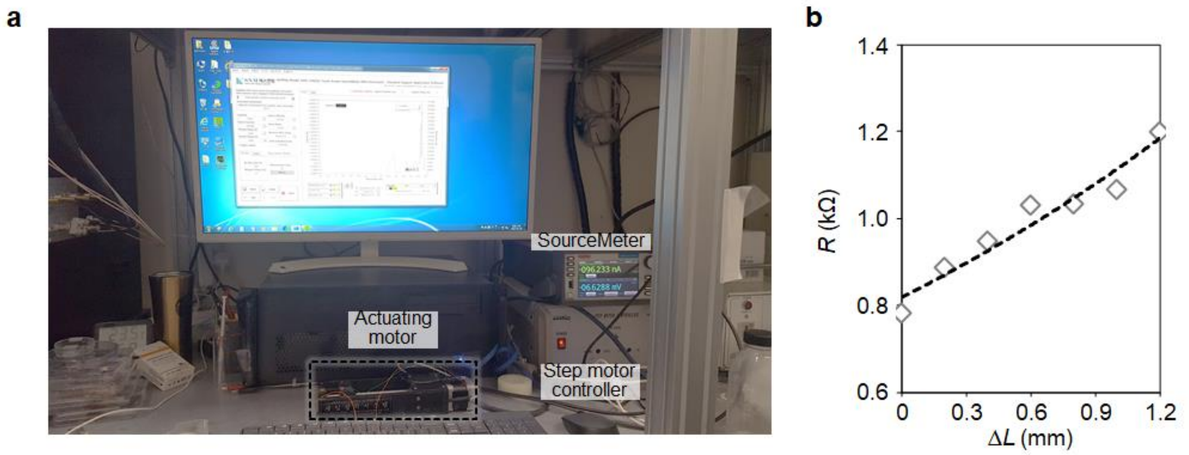

Figure S12. The resistance (*R*) measurement of CNT/graphene/SEBS composite sensor. a) Resistance measurement setup consisting of source meter, step motor controller, and actuating motor. b) Resistance measurement of the composite sensor according to the difference in length (ΔL).

As shown in Figure S12a, we prepared a Keithley 2450 Source meter, a step motor controller (SMC-100, ECOPLA), and an actuating motor (Jaeil optical system) to monitor resistance values according to ΔL. The CNT/graphene/SEBS composite sensor was cut into 20-mm length and 5-mm width, and we attached it onto the actuating motor. The resistance value was measured through a source meter connected to both ends of the sensor while gradually changing ΔL (Figure S12b).

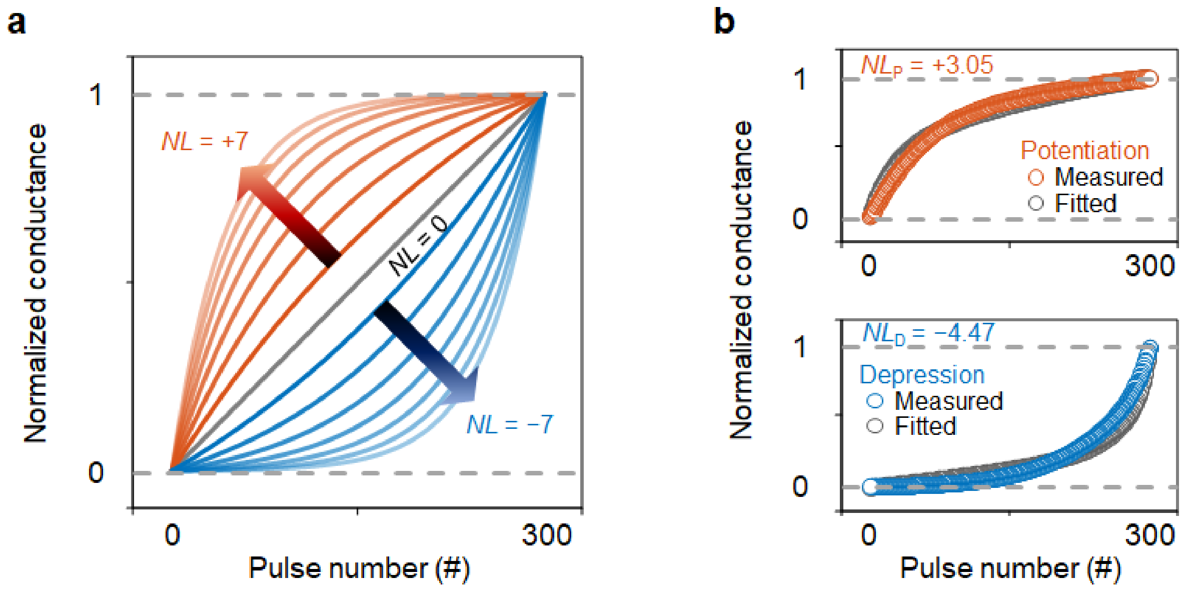

Figure S13. *NL* analysis of LTP/D characteristic curves. a) Normalized G_{LTP}/G_{LTD} characteristic curves with respect to *NL* ranging from 0 to 7. b) Measured and fitted curves in LTP (upper panel) and LTD (lower panel) regions, where $NL_P = +3.05$ and $NL_D = -4.47$.

There are several methods to evaluate the *NL* of the LTP/D characteristic curve.[S3-S5] Among them, we chose a method to tune A_P and A_D for finding the G_{LTP}/G_{LTD} curves best matched to the measured LTP/D curves.[S6] The G_{LTP}/G_{LTD} curve model with the number of pulses (P) is represented as the following equations:

$$G_{LTP} = B_P \cdot (1 - \exp(-P/A_P)) + G_{min}, \quad (3)$$

$$G_{LTD} = -B_D \cdot (1 - \exp((P - P_{max})/A_D)) + G_{max}, \quad (4)$$

$$B_{P,D} = (G_{max} - G_{min})/(1 - \exp(-P_{max}/A_{P,D})). \quad (5)$$

where G_{LTP} and G_{LTD} are the conductance values for LTP and LTD, respectively. G_{max}, G_{min}, and P_{max} are the measured data that represent the maximum conductance, minimum conductance, and maximum pulse number, respectively. $B_{P,D}$ is a fitting constant to normalize the conductance range. A_P and A_D are parameters that determine the nonlinearities of the weight update in the LTP and LTD regions, which are directly related to the *NL* values. The G_{LTP}/G_{LTD} curves with respect to the *NL* ranging from 0 to 7 are displayed in Figure S13a. By adjusting the A_P and A_D values, the G_{LTP}/G_{LTD} curves are fitted to the measured LTP/LTD curves, and accordingly the *NL* values are determined (Figure S13b).

Table S1. Comparison of proposed vertical synaptic device and other synaptic devices in terms of learning accuracy predicted by NeuroSim+ MNIST MLP simulator.[S3]

	**Ag:a-Si**	**TaO_x/HfO_x**	**PCMO**	**AlO_x/HfO_2**	**GST PCM**	**Epi-RAM**	**HZO FeFET**	**This work**	**Ideal**
**Nonlinearity (P/D)**	2.4/−4.88	0.04/−0.63	3.68/−6.76	1.94/−0.61	0.105/2.4	0.5/−0.5	1.75/1.46	**3.68/−4.88**	0/0
**Asymmetry**	7.28	0.67	10.44	2.55	-2.295	1.0	0.29	**8.56**	0
**ON/OFF ratio**	12.5	10	6.84	4.43	19.8	50.2	45	**31.3**	>10
**# of conductance states**	97	128	50	40	100–120	64	32	**300**	>64
**Cycle-to-cycle variation**	3.5%	3.7%	<1%	5%	1.5%	2%	<0.5%	**<1%**	0%
**Online learning accuracy**	~72%	~80%	~33%	~20%	89%	92%	88%	**87.2%**	93.5%
**Reference**	S7	S8	S9	S10	S11	S12	S13	**-**	-

Table S2. Synaptic characteristics for recognition tasks

Device	Non-linearity of LTP (NL_P)	Non-linearity of LTD (NL_D)	G_{max}/G_{min} ratio	Number of conductance states	R_{on} [Ω]	Cycle to cycle variation
Ideal synapse device	0	0	>10	>128	100 K	<1%
Vertical synapse device	3.68	−4.88	31.3	300	104 M	~1%

References

[S1] S. Seo, B.-S. Kang, J.-J. Lee, H.-J. Ryu, S. Kim, H. Kim, S. Oh, J. Shim, K. Heo, S. Oh, J.-H. Park, *Nat. Commun.* **2020**, *11*, 3936.

[S2] Y. Choi, S. Oh, C. Qian, J.-H. Park, J. H. Cho, *Nat. Commun.* **2020**, *11*, 4595.

[S3] P.-Y. Chen, X. Peng, S. Yu, in *2017 IEEE Int. Electron Devices Meet.*, IEEE, **2017**, pp. 6.1.1-6.1.4.

[S4] C. Sen Yang, D. S. Shang, N. Liu, E. J. Fuller, S. Agrawal, A. A. Talin, Y. Q. Li, B. G. Shen, Y. Sun, *Adv. Funct. Mater.* **2018**, *28*, 1.

[S5] L. Yin, C. Han, Q. Zhang, Z. Ni, S. Zhao, K. Wang, D. Li, M. Xu, H. Wu, X. Pi, D. Yang, *Nano Energy* **2019**, *63*, 103859.

[S6] S. Gandla, M. Naqi, M. Lee, J. J. Lee, Y. Won, P. Pujar, J. Kim, S. Lee, S. Kim, *Adv. Mater. Technol.* **2020**, *5*, 1.

[S7] S. H. Jo, T. Chang, I. Ebong, B. B. Bhadviya, P. Mazumder, W. Lu, *Nano Lett.* **2010**, *10*, 1297-1301.

[S8] Y.-F. Wang, Y.-C. Lin, I.-T. Wang, T.-P. Lin, T.-H. Hou, *Sci. Rep.* **2015**, *5*, 10150.

[S9] S. Park, A. Sheri, J. Kim, J. Noh, J. Jang, M. Jeon, B. Lee, B. R. Lee, B. H. Lee, H. Hwang, *IEEE IEDM*, **2013**.

[S10] J. Woo, K. Moon, J. Song, S. Lee, M. Kwak, J. Park, H. Hwang, *IEEE Electron Device Lett.*, **2016**, *37*, 994-997.

[S11] D. Kuzum, R. G. D. Jeyasingh, B. Lee, H.-S. P. Wong, *Nano Lett.* **2011**, *12*, 2179-2186.

[S12] S. Choi, S. H. Tan, Z. Li, Y. Kim, C. Choi, P.-Y. Chen, H. Yeon, S. Yu, J. Kim, *Nat. Mater.* **2018**, *17*, 335-340.

[S13] M. Jerry, P.-Y. Chen, J. Zhang, P. Sharma, K. Ni, S. Yu, S. Datta, *IEEE IEDM*, **2017**.”
